# The effect of social identity on objective and perceived social support in groups

**DOI:** 10.1111/bjso.70130

**Published:** 2026-09-08

**Authors:** Julia Heimrich, Diana Usmanova, Nina Mareen Junker, Rolf van Dick, Jan Alexander Häusser

**Affiliations:** ^1^ Department of Social Psychology Justus‐Liebig‐University Gießen Giessen Germany; ^2^ Department of Social Psychology Goethe‐University Frankfurt Frankfurt am Main Germany; ^3^ Department of Psychology University of Oslo Oslo Norway; ^4^ Department of Psychology Lingnan University Lingnan Hong Kong

**Keywords:** mutual social support, objective social support, perceived benevolence, perceived provided social support, perceived received social support, social identity

## Abstract

The social identity approach posits that social identity fosters mutual social support within groups. However, prior research has relied almost exclusively on individual‐level self‐report measures, neglecting the mutuality of support and the group as a unit of analysis. This pre‐registered one‐factorial online experiment with three‐person groups (*N* = 306 group members; *N* = 102 groups) examined how social identity shapes objective mutual support, perceived provided and received support and the perceived benevolence of received support. Following a pre‐tested identity manipulation (*N* = 120), groups completed a video‐recorded workplace simulation. Transcripts were blind‐coded. Groups in the social identity condition engaged in significantly more objective mutual social support than participants in the personal identity condition. Social identity (vs. personal identity) did not affect perceived support but increased perceived benevolence, in terms of assuming underlying motives. These findings highlight the importance of distinguishing among objective mutual social support at the group‐level, perceived social support and the interpretation of social support to understand how shared social identity shapes the social support process.

More than two decades of research on the Social Identity Approach to Health and Well‐being (Haslam et al., 2009) highlight the crucial role of the psychological partnership between social identity and social support (Haslam et al., 2012; Häusser et al., 2020). The Social Identity Approach (comprising social identity theory and self‐categorization theory; Tajfel & Turner, 1979; Turner et al., 1987) states that sharing a social identity increases social support among group members, as it is grounded in shared goals, norms and values (Cigognani et al., 2012; Hogg & Reid, 2006; Ketturat et al., 2016; van Dick & Haslam, 2012). Consequently, social support has been identified as a key pathway through which a shared social identity exerts its beneficial effects on health and well‐being (Haslam et al., 2009; Jetten et al., 2012; van Dick & Haslam, 2012).

Although this relationship has been tested in earlier research (e.g. Avanzi et al., 2015; Frenzel et al., 2023; Junker et al., 2019), these studies have theoretical and empirical constraints that the present research seeks to address. First, a core proposition of the Social Identity Approach to Health and Well‐being is that a shared social identity fosters *mutual* social support among groups (Haslam et al., 2009; Jetten et al., 2012). As Häusser et al. (2020) note, there is a disconnect between this theoretical prediction and its prior empirical tests. Whereas the theoretical argument concerns sharing a group identity and mutual support (i.e. a group‐level process), the link between social identity and social support has almost invariably been examined at the individual‐level, focusing on the association between individuals' identification with a team and their perceptions of provided and received support (e.g. Avanzi et al., 2015; Junker et al., 2019; Walsh et al., 2015; see van Dick et al., 2018 for a discussion). Importantly, this does not imply that the association between social identity and social support should exclusively be studied at the group‐level. Instead, Häusser et al. (2020) proposed that—in addition to studying the effect of shared social identity on mutual social support as a group‐level association—the perception and interpretation of support (i.e. attribution and appraisal) are individual‐level processes that are shaped by a group member's own identification (rather than other members' identification). To capture this dynamic and more thoroughly test the central proposition of the Social Identity Approach to Health and Well‐Being, we therefore provide a first test of the effect of shared identity on mutual social support.

Second, prior research on the social identity‐social support link has relied primarily on self‐report measures that capture social support (e.g. Haslam et al., 2005; Häusser et al., 2023). Although perceptual measures of social support capture valuable subjective experiences, perceived social support is inherently retrospective and likely shaped by interpretations (Lakey & Cohen, 2000). Hereby, a shared social identity might contribute to such identity‐based perceptions (Tajfel & Turner, 1979). Specifically, it remains unclear whether a shared social identity objectively increases provided and received social support or merely leads group members to perceive behaviours by their peers as more supportive. In line with this concern, perceived and objective support are distinct, with only moderate correlations (Barrera, 1986), suggesting that people might feel supported even in the absence of actual social support or, conversely, might actually be supported without recognizing or valuing it. Testing the effect of social identity on objective social support therefore allows for a more comprehensive assessment of social support within groups.

Third, previous measures of perceived social support vary considerably in the sources, interactions and forms of support they capture, ranging from retrospective reports over extended periods (e.g. ‘In the past four weeks…’; Wills & Shinar, 2000) to more general assessments of currently felt support (e.g. ‘I feel supported’; Haslam et al., 2005, 2018). What most of these measures have in common is that they aggregate across various sources (e.g. a number of unspecified co‐workers) and interactions, and are therefore not tied to a specific group interaction, for instance a specific task with a defined number of co‐workers. Such measures thus lack the specificity needed to disentangle perceptions (i.e. interpretations) from behaviours within particular group interactions. Moreover, research on social identity and perceived social support in teams has predominantly examined perceptions of *received* support (‘My team supported me’; Haslam et al., 2005; Häusser et al., 2023; Junker et al., 2019), largely neglecting perceptions of *providing* support (‘I supported my team’; Shakespeare‐Finch & Obst, 2011). The few studies that assessed both facets in relation to social identity were correlational and relied on retrospective self‐reports (e.g. Hutchison et al., 2018; Steffens, Jetten, et al., 2016). By assessing both perceived received and provided support following the same standardized group interaction, we can examine how social identity simultaneously influences these complementary elements of social support, allowing for a more fine‐grained examination of how social identity shapes support processes.

Fourth, prior research indicates that the meaning attributed to social support may depend on the social identity context. For example, prior studies showed that social support during a stressful event reduced stress reactions only when group members shared a social identity (Frisch et al., 2014). Whether support is effective may thus depend not only on how much support group members perceive they exchange, but also on *why* they believe it is given and the motives they attribute to it (strikingly, in the Frisch et al., 2014 study, support was standardized by the experimental design). Yet, prior research has focused almost exclusively on the *quantity* (i.e. whether and how much support is perceived to occur), while largely neglecting the interpretation of the underlying motive. We argue that the perceived benevolence of social support (i.e. the extent to which an individual is believed to want to do good to another person, aside from egocentric motives; Mayer et al., 1995) represents a central feature of the identity‐support link (Häusser et al., 2020, see also Semmer et al., 2008). Theoretical accounts suggest that a shared social identity creates beliefs that ingroup members act to benefit the collective rather than pursue self‐interest (Borinca et al., 2019; Turner et al., 1987). Accordingly, a shared social identity should not only increase objective and perceived provided and received social support within a group but also influence whether group members interpret the support they receive as more benevolent.

Building on the Social Identity Approach, the present study uses a pre‐registered one‐factorial experimental design to investigate the causal effects of social identity on objective and perceived mutual social support, as well as the corresponding perception of benevolence within groups in a standardized interactive group task. To this end, we manipulated identity salience (social identity condition vs. personal identity condition) in ad hoc groups—pre‐tested in a pre‐registered pilot study—prior to a group task, during which we measured various aspects of objective and perceived social support.

## SOCIAL IDENTITY AND SOCIAL SUPPORT

The Social Identity Approach (Tajfel & Turner, 1979; Turner et al., 1987) provides a framework for understanding how individuals define themselves in relation to social groups. According to this approach, individuals derive part of their self‐concept from their membership in social groups, and this social identity shapes how they perceive, evaluate and behave towards others. Group memberships become psychologically meaningful when individuals self‐categorize as members of the group and develop an identification with it, thereby internalizing group goals, norms and values (Turner et al., 1994).

The strength of social identification varies as a function of situational cues, comparative contexts and the perceived relevance of the group to the self (Hogg & Reid, 2006). As a result, a shared social identity can be (experimentally) induced or intensified by making a particular group membership salient, with downstream implications for how individuals think, feel and behave within group interactions (Frisch et al., 2014; Häusser et al., 2012; Levine et al., 2005). Extending this foundation, the Social Identity Approach to Health and Well‐Being emphasizes that a shared social identity has beneficial effects on mental and physical health outcomes (Haslam et al., 2009; Jetten et al., 2012; see Steffens et al., 2017 and Cruwys et al. 2025 for meta‐analyses).

This framework further argues that a central mechanism by which shared social identity affects health and well‐being is social support. Specifically, Haslam et al. (2018; see also Jetten et al., 2017) proposed in their social support hypothesis that: ‘When, and to the extent that, people define themselves in terms of shared social identity, they will (a) expect to give each other support, (b) actually give each other support, and (c) construe the support they receive more positively’ (p. 30). Häusser et al. (2020) extended this hypothesis by emphasizing the distinction between individual‐ and group‐level processes, arguing that objective mutual social support is a group‐level phenomenon that arises from a shared social identity and the identification of all group members with the group. In contrast, the perception and interpretation of social support operate at the individual level, where an individual's own identification with the group (rather than the sharedness or group's average identification) should be the more relevant predictor. The meaningfulness of the group‐level as a level of analysis in social identity research is also supported by meta‐analytic evidence demonstrating that group identification (the aggregated identification of individual group members) has positive effects on well‐being beyond individual identification (Steffens et al., 2017).

### Social identity and objective social support within a group

While the association between social identification and social support has been extensively tested (e.g. Drury et al., 2016; Haslam et al., 2005; Häusser et al., 2023; Junker et al., 2019; Sidorenkov & Borokhovski, 2021; Walsh et al., 2015), these studies almost exclusively relied on self‐reported perceptions of received support. However, the Social Identity Approach implies behavioural consequences of a group (as a whole) (Tajfel & Turner, 1979; Turner et al., 1987). Therefore, it remains unclear whether a shared social identity increases objective mutual social support—observable interpersonal behaviours such as providing or receiving information, assistance and encouragement—during group interactions, merely shapes individual perceptions of support or affects both.

A shared social identity shifts an individual's perspective from oneself to the group, increasing the salience and importance of group‐relevant outcomes (Turner et al., 1987). Consequently, shared social identity should motivate behaviours that support the group's functioning to achieve these collective goals and outcomes (Neville & Reicher, 2011; Reicher & Haslam, 2010; Tyler & Blader, 2001). Supporting fellow group members during an interaction should align with acting in the interest of the group as a whole. Preliminary research on intergroup helping supports the argument that social identity can shape objective social support. In two experiments, Levine et al. (2005) demonstrated that individuals are more likely to help a stranger in distress—coming to the aid of someone who has taken a fall—when that person is perceived as an ingroup member (a fan of the same football team, identified by wearing the club's jersey) than an outgroup member (a fan of a rival team, wearing that club's jersey). Further supporting the importance of a shared social identity, they showed that making a more inclusive shared social identity salient (being a football fan in general and wearing the English national team's jersey) extended helping behaviours to individuals who had previously been categorized as outgroup members (being a fan of a specific football team). Critically, with only 45 participants in Study 1 and 32 participants in Study 2—each distributed across three experimental conditions—both studies were severely underpowered by today's standards. Moreover, Levine et al. (2005) focused on individuals helping in emergency situations characterized by asymmetric roles between victims and bystanders, rather than on mutual social support among equal group members in a shared task. Complementary evidence that shared social identity shapes support at the level of observable behaviour comes from the BBC Prison Study—an experimental case study (Haslam & Reicher, 2006; Reicher & Haslam, 2006). Mutual social support was expected and provided among participants who developed a shared social identity (the prisoners), enabling them to coordinate collectively, but was not observed among those who did not develop a shared social identity (the guards). However, this work was not designed as a controlled experimental test of causal effects, underscoring the value of the present approach.

Addressing these limitations requires a shift from examining individual helping towards examining group‐level processes. If shared social identity motivates individuals to prioritize collective goals and group welfare (Haslam et al., 2005), this motivation should thus be reflected in mutual social support at the group‐level. Taken together, these considerations lead to the following hypothesis (tested on the group‐level):

In the social identity condition, objective mutual social support within a group will be higher than in the personal identity condition.^1^

### Social identity and perceived received and provided support

As stated above, the theoretical basis for the expected link between social identity and social support rests on the concept of mutual social support (van Dick & Haslam, 2012). Extending this idea to the individual‐level means clearly separating perceptions of received and provided support. In line with this assumption, Gleason et al. (2003) emphasize that reciprocity in supportive exchanges is key, suggesting that support is not provided or received in isolation but unfolds as a reciprocal process. Within group interactions, group members are thus simultaneously potential providers and recipients of social support, and when a social identity is salient, support is embedded within a collective ‘we’‐frame, making it normative and meaningful for all group members (Yalcin & Hopkins, 2025).

However, research has predominantly measured perceived received support and has given little attention to perceptions of providing support, despite evidence that provided and received social support constitute distinct facets of social support (Brown et al., 2003; Häusser et al., 2020; Mbuthia et al., 2022; Norris et al., 2001; Nurullah, 2012; Song et al., 2014). The few studies that jointly examined both facets in the context of social identity exclusively relied on correlational, self‐report designs. These studies showed, for instance, that group identification predicts the support members report providing as well as receiving in recovery groups (Hutchison et al., 2018) and among retirees (Steffens, Jetten, et al., 2016). In a qualitative study, Hartley et al. (2022) similarly found that a shared identity shapes the provision and receipt of support within teams. As such, designs assess support retrospectively and outside the interaction in which it occurs, they cannot establish whether a shared social identity causally predicts provision and receipt of social support as it unfolds. Thus, we predict that social identity should foster both the perception of received and provided social support in group interactions (tested at the individual‐level).

In the social identity condition, (a) provided and (b) received social support will be perceived as higher than in the personal identity condition.

### Social identity and perceived benevolence of social support

As receiving social support is not merely an individual act of helping but a group‐relevant behaviour, it reflects concern for fellow group members and commitment to the collective (Hüffmeier et al., 2014). This implies that social support among group members can be interpreted as driven by benevolent motives. Perceived benevolence captures this interpretation and concerns the motive that recipients ascribe to an act of support, rather than whether the act occurred. Individuals might therefore perceive a given act as clearly supportive yet remain uncertain whether it stems from genuine concern or egoistic motives.

Haslam et al. (2012) state that the interpretation of received social support depends on the identity‐based relationship between the provider and recipient of support. That is, sharing a social identity should not only increase the likelihood of mutual social support but also shape how group members interpret such support. Likewise, Häusser et al. (2020) propose that a shared social identity should lead to a more benevolent interpretation of received social support. That is, when social identity is shared, individuals should be more likely to attribute prosocial motives to the support provided by fellow group members, perceiving their behaviour as serving a mutual goal and driven by ingroup‐enhancing motives. Without a shared social identity, no identity‐based positive relationship or mutual interests exist and individuals should therefore be more likely to question the motives underlying support, perceiving them to be less benevolent (Zagefka et al., 2023).

Empirical evidence is consistent with this idea. McKimmie et al. (2020) provide initial, indirect evidence for the positive case, showing that participants perceived other group members' behaviour as more supportive when a shared social identity was salient. Conversely, support offered outside a shared identity may be experienced as patronizing or as an assertion of the provider's power rather than as a genuinely benevolent act (Nadler, 2010). For instance, Halabi et al. (2016) demonstrated that assistance offered by a high‐status outgroup is more likely to be interpreted by members of a low‐status group as a means of achieving dominance and reinforcing dependency. In sum, we expect social identity to influence individuals' perceptions of the benevolence of social support from their group members (tested at the individual‐level).

In the social identity condition, social support will be perceived as more benevolent than in the personal identity condition.

## METHOD

We conducted a one‐factorial (personal vs. social identity) online experiment to test our hypotheses. All measures, manipulations, analyses and data/participant exclusions are reported in the manuscript, in the Supporting Information, or are uploaded to a public repository (https://osf.io/y6utk/overview?view_only=c658b464beaa4144b9e3843c1fc96dfb). An a‐priori power analysis for the group‐level comparison showed that our aimed *N* is sufficient to identify a medium effect size of *d* = .50, with a power of .85 and an alpha of .05 for our group‐level hypothesis (H1). Sensitivity analysis indicated that the sample size is also sufficient to detect small‐to‐medium effect sizes (*d* = .30) with a power of .85 and *α* = .05 for the individual‐level hypotheses H2 and H3. The study was pre‐registered (https://aspredicted.org/fsp6‐xphx.pdf), approved by the local ethics committee [Justus‐Liebig University, Giessen] and participants gave their informed consent. The authors acknowledge the use of Rev's AI transcription service (accessed March 2025) to generate initial transcripts of the audio data. All AI‐generated transcripts were carefully reviewed, corrected where necessary and verified by the authors before being used for analysis.

### Sample

Initially, 312 participants took part in the study. Six participants (and consequently two groups for the group‐level analyses) were excluded: one group was excluded because a member completed the personal identity manipulation task (see below) despite being assigned to the social identity condition, and one group was excluded because a participant had taken part in the study twice. Therefore, the final sample consisted of 306 university students, distributed across 102 groups (51 per experimental condition), each with three participants. Because two video recordings were lost due to technical failure, the sample for the group‐level analyses of objective mutual social support (H1) was reduced to 100 groups (50 per experimental condition).

Seventy‐five participants were recruited through the online panel Prolific, while the remaining 231 participants were recruited via university mailing lists. The sample comprised 189 women (61.8%), 112 men (36.6%), four participants who identified as diverse (1.3%), and one participant who did not disclose their gender (0.3%). Participants ranged in age from 18 to 44 years (*M* = 24.24, *SD* = 4.02).

### Procedure

The study was conducted online via Zoom,^2^ with participants and the experimenter present together in the same virtual meeting room. Participants were requested to switch their video and audio on throughout the session, allowing them to see and hear one another, but were instructed to remain silent unless explicitly prompted by the experimenter, when encountering technical difficulties or during the group discussion. Experimental sessions were randomly assigned to either the social identity or the personal identity condition. We invited three previously unacquainted participants to each experimental session, offering a 25€ incentive and the opportunity to win an additional 20€. The study lasted approximately 70 min. The experimenter sent the virtual backgrounds (a shared virtual background for the social identity condition and individual virtual backgrounds for the personal identity condition) before the experimental sessions. Upon arrival in the virtual meeting room, participants were instructed to turn on their camera and audio. After a brief round of introductions (name, age and field of study), we manipulated their social identity. Following the manipulation, participants completed a first survey assessing identification with the group as a manipulation check. Participants then engaged in a 25‐min group task in which they assumed the role of employees at a fictional airline, ‘Corgi Atlantic’, and worked collaboratively on a series of task‐related activities. Using a shared file, they worked on six pre‐assigned tasks that required problem‐solving. Each participant was assigned to two tasks—one where they lacked information that another group member had access to, and one where they had access to information that another group member lacked. Crucially, participants were not informed about this interdependent task structure. For instance, one task required participants to conduct a cost analysis of new flight routes, considering factors such as fuel, personnel and airport fees, while another task involved creating a weekly flight schedule that accounted for destinations, aircraft availability and staffing. The complete task descriptions are provided in Appendix A. Because all participants worked simultaneously in a shared document, they could either actively ask for or offer social support or independently search for relevant information within it. The group task was video‐recorded. After the group task, the second survey was administered, assessing perceived social support (provided and received), as well as the perceived benevolence of social support.^3^ Finally, participants were debriefed about the study's aims.

### Experimental manipulation

To manipulate social identity, we adapted established procedures for inducing a shared social identity (Häusser et al., 2012; see also Frisch et al., 2015, for a video tutorial) to an online setting.

**FIGURE 1:**
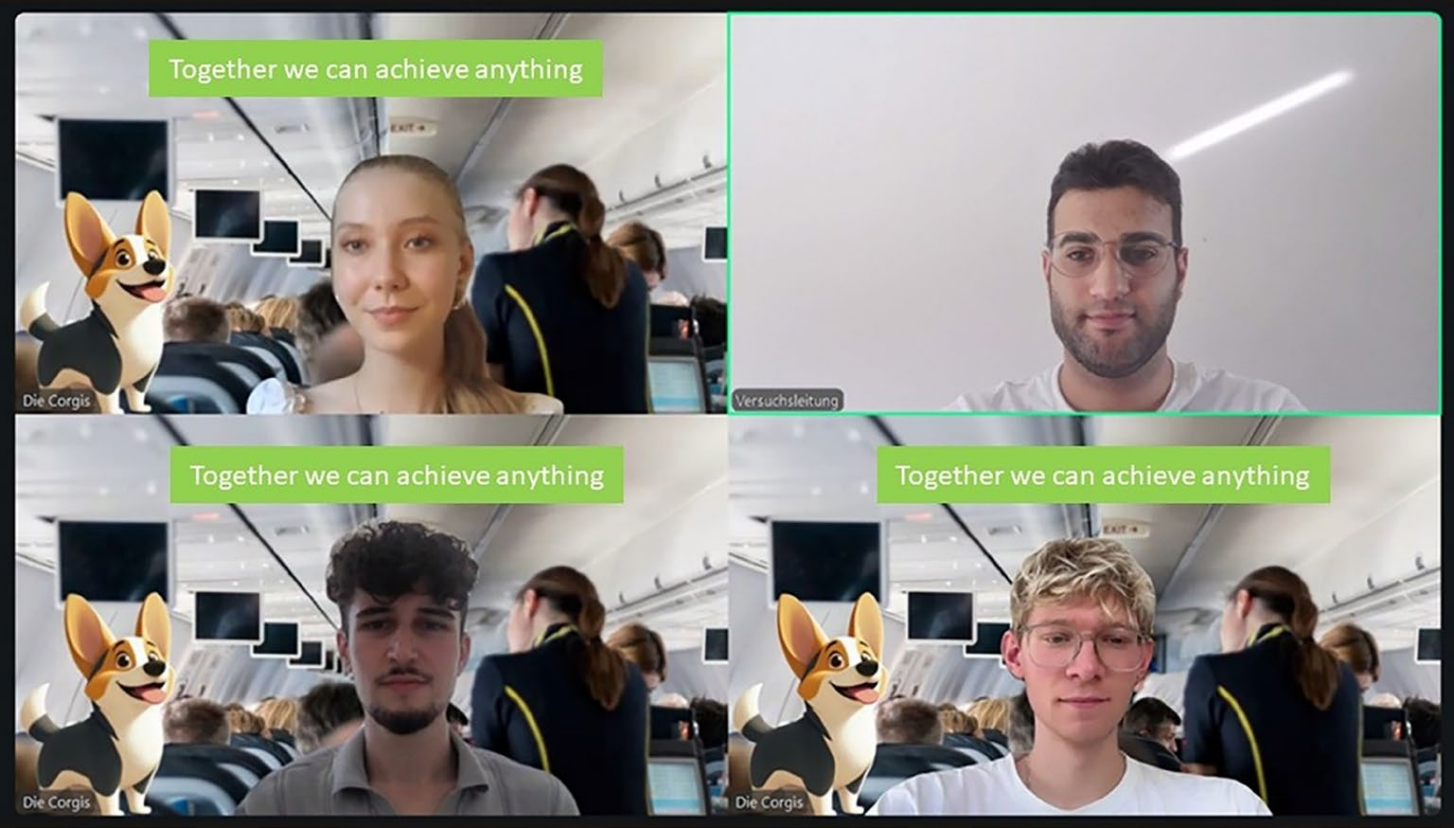
Social identity condition: Shared background, team slogan, team name and team logo (experimenter in the upper‐right corner).

In the *social identity condition*, participants were treated as a group. (1) All instructions were delivered collectively, addressing participants as members of a unified team (‘Your team will…’). (2) Each group received a team name (‘The Corgis’) that they were instructed to use as their name label in Zoom. (3) Participants received an identical virtual background and a shared team slogan. (4) Additionally, they received a specific sticker (an animated Corgi dog) which represented their team's name (see Figure 1 for the experimental manipulation). (5) To further emphasize social identity, participants were tasked with individually brainstorming three things that can be done to improve customer satisfaction in the event of flight delays. Participants wrote their ideas in our online survey and were informed that the group with the best collective ideas would receive an additional 20€ in compensation. (6) Finally, participants should list three characteristics they believed they shared with their fellow group members.

**FIGURE 2:**
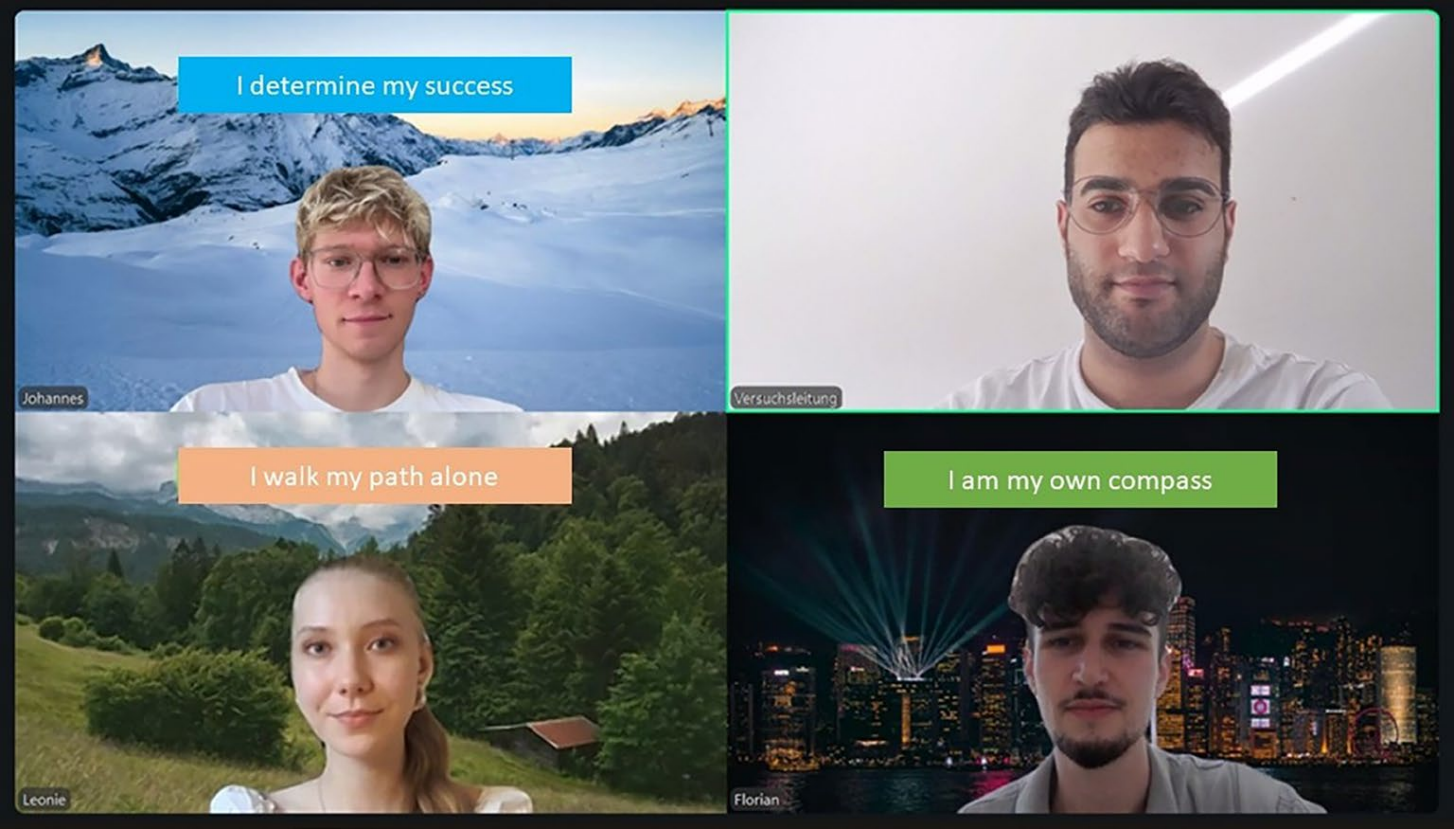
Personal identity condition: Individual background, individual slogan, given name (experimenter in the upper‐right corner).

In the *personal identity condition*, the focus was on inter‐individual differences and personal characteristics. (1) Instructions were delivered to participants individually, emphasizing their unique contributions (‘You will…’). (2) Participants kept their personalized name in Zoom and (3) received individual virtual backgrounds and slogans (see Figure 2). (4) During the brainstorming task, participants were informed that the prize would be awarded to the individual with the best ideas rather than to the group as a whole. (5) Finally, participants were told that they should list three characteristics that distinguished them from the other group members.

#### Pilot study

To evaluate whether our experimental procedure effectively manipulated social versus personal identity in an online setting, we conducted a pre‐registered pilot study (https://aspredicted.org/g6p7‐2pmj.pdf). We expected that participants' identification with the group would be higher in the social identity condition than in the personal identity condition.

A total of 120 individuals participated, recruited via an online panel. An a‐priori power analysis showed that this *N* is sufficient to identify a medium effect size of *d* = .50, with a power of .85 and an alpha of .05 (one‐tailed). The sample included 75 men (62.5%), 43 women (35.8%) and two participants who identified as divers (1.7%). Participants were on average 28.87 years old (*SD* = 5.23). All analyses were conducted in SPSS v.29. We tested our hypothesis using an independent sample *t*‐test. As expected, participants in the social identity condition reported significantly higher levels of identification with their group (*M* = 3.55, *SD* = .64) than participants in the personal identity condition (*M* = 3.09, *SD* = 0.87), *t*(118) = 3.32, *p* < .001, 95% CI [0.19, 0.74], *d* = 0.61.

### Measures

*Identification with the group (manipulation check)* was measured using four items developed by Doosje et al. (1995). The participants indicated to which degree they agreed with four items (sample item: ‘I identify with the workgroup.’) on a scale from 1 = *Fully disagree* to 5 = *Fully agree*. Cronbach's *α* was .76.

*Perceived provided social support* was measured using five items adapted from Boyar et al. (2014) to capture specific supportive behaviours during the group task (e.g. ‘I listened to the problems my group members had with the group task’). Participants rated to what extent they agreed with each item on a scale from *1 = Do not agree at all* to *7 = Fully agree*.^4^ Cronbach's *α* was .86.

*Perceived received social support* was also measured using five items adapted from Boyar et al. (2014). Participants indicated to what extent they agreed with the items (sample item: ‘My workgroup shared work ideas with me.’) on a scale from *1 = Do not agree* at all to *7 = Fully agree*. Cronbach's *α* was .89.

*Perceived benevolence of support* was measured using three self‐developed items. Participants indicated the extent to which they agreed with the statements (‘The other group members helped me with the group task to show that they believe in me’; ‘The other group members helped me with the group task because I really mattered to them’ and ‘The other group members helped me with the group task because they wanted to support me in completing the task as well as possible’) on a scale from *1 = Fully disagree* to *5 = Fully agree*. Cronbach's *α* was .71.

*Objective mutual social support* was assessed through behavioural coding of video transcripts of the group task by two independent, trained coders who were blind to the study conditions. Inter‐rater reliability for mutual social support was .91.

A structured coding scheme was developed to identify and quantify social support during the group discussion (see public repository for the coding scheme). Mutual social support was coded using two main categories: support provided without a request and support received after a request. See Table 1 for illustrative examples from the transcripts. We further coded the type of support, that is, whether it was informational (e.g. sharing relevant information or ideas), instrumental (e.g. providing hands‐on assistance with a task) or emotional (e.g. encouraging others). Since we had no specific hypotheses for the type of support and because case numbers in subcategories were relatively small, we aggregated all types of support into a single measure of mutual social support.

**TABLE 1:** Illustrative examples for social support actions during the group task.

Mutual social support during the group task	Example
Providing informational support without request	‘So, I've finished my first task. I do not know if you need this, but I have now divided up the shifts in number four. It is now in the table. Maybe one of you will need it’
Providing instrumental support without request	‘If someone needs help, tell me’!
Providing emotional support without request	‘It is all good, we are in this together’
Received informational support after request	‘I am missing some information for my part. Does anyone have information on the costs for the airline's destinations’?
Received instrumental support after request	‘Does anyone have a moment for the last task? I think I might have made a mistake there’

### Analyses

The hypotheses were tested using a single‐factor between‐subjects design (identity: social vs. personal). We prepared the data in SPSS v.29 and tested our hypotheses in Mplus 8.8 (Muthén & Muthén, 1998–2017). As pre‐registered, hypotheses were tested one‐tailed, with effects considered statistically significant at *p* < .05. All outcomes were estimated within a single model, allowing their residuals to covary.

For Hypothesis 1, objective mutual social support was operationalized as the number of supportive interactions in each team during the group task. Repeated references to the same support issue were coded only once, unless a new support interaction occurred and the two coder ratings were averaged prior to analysis. Although each supportive act was performed by an individual, these acts unfold in the interaction between (at least two) group members and jointly constitute the reciprocal exchange that characterizes the group as a whole, which is why mutual social support is best conceptualized and analysed at the group rather than the individual‐level (Häusser et al., 2020; van Dick & Haslam, 2012). Objective mutual social support (H1) was therefore modelled as a group‐level (Level‐2) outcome, regressed on the experimental condition (*n* = 50 groups per condition).

Hypotheses 2 and 3 concern individual‐level perceptions (perceived provided support, perceived received support and perceived benevolence) assessed from participants nested within groups. To align the analysis with this nested structure, in which the experimental manipulation varied at the group‐level while the outcomes were measured at the individual‐level, we tested these hypotheses using multilevel models in Mplus. We specified a two‐level random‐intercept model with individuals at Level 1, nested in groups at Level 2. The experimental condition was, again, entered as a Level‐2 (between‐group) predictor of all outcomes.

All analyses were conducted using a Bayesian estimator. We chose Bayesian estimation because it is less dependent on the assumption of normally distributed data than maximum likelihood estimation (Kaplan & Depaoli, 2012), provides more stable parameter estimates when the number of clusters is relatively small (Asparouhov & Muthén, 2021), and tends to converge more readily in multilevel models, where maximum likelihood estimation can become computationally demanding (Muthén & Asparouhov, 2012).

## RESULTS

### Descriptive results and manipulation check results

Means, Standard Deviations, ICCs and Correlations are found in Table 2. Intraclass correlations indicated substantial between‐group variance for all three outcomes (.35 for perceived provided support, .38 for perceived received support and .19 for perceived benevolence), confirming that the nested structure needed to be modelled. We first tested the effectiveness of our manipulation in producing social versus personal identity salience before proceeding with our hypotheses testing. Participants in the social identity condition reported significantly higher levels of identification with the group (*M* = 3.32, *SD* = 0.75) than those in the personal identity condition (*M* = 3.08, *SD* = .72), *b* = 0.24, *SD* = 0.09, *p* = .004 (one‐tailed), 90% CI [0.09, 0.39], *β* = .60.

**TABLE 2:** Means, standard deviation and correlation of all variables.

	Identity	*N*	*M*	ICC	1	2	3	4
Individual‐level								
1. Identification	Social	153	3.32 (.75)	.02		.10	.17**	.31**
	Personal	153	3.08 (.72)					
2. Perceived provided support	Social	153	4.76 (1.60)	.35			.46**	.45**
	Personal	153	4.47 (1.70)					
3. Perceived received support	Social	153	5.10 (1.55)	.38				.61**
	Personal	153	4.94 (1.68)					
4. Benevolence	Social	153	3.04 (.81)	.19				
	Personal	153	2.83 (1.00)					
Group‐level								
1. Identification (aggregated identification)	Social	51	3.32 (.42)	–		.11	–	–
	Personal	51	3.08 (.42)					
2. Mutual social support	Social	50	2.20 (1.13)	–			–	–
	Personal	50	1.72 (1.03)					

*Note*: **p < .01.

### Results of hypothesis testing

Supporting Hypothesis 1, objective mutual social support during the group task was stronger for groups in the social identity condition (*M* = 2.20, *SD* = 1.13) than it was in the personal identity condition (*M* = 1.72, *SD* = 1.03), *b* = 0.56, *SD* = 0.23, *p* = .007 (one‐tailed), 90% CI [0.19, 0.94], *β* = .24.

Hypothesis 2 predicted that both (a) provided and (b) received support would be perceived as higher by participants in the social identity condition than they would be in the personal identity condition. Neither adifference between the social identity condition (*M* = 4.76, *SD* = 1.60) and the personal identity condition (*M* = 4.47, *SD* = 1.70) were found for perceived provided support *b* = 0.29, *SD* = 0.26, *p* = .133 (one‐tailed), 90% CI [−0.14, 0.73], *β* = .14, nor a difference between the social identity condition (*M* = 5.10, *SD* = 1.55) and the personal identity condition (*M* = 4.94, *SD* = 1.68) for perceived received support *b* = 0.16, *SD* = 0.26, *p* = .262 (one‐tailed), 90% CI [−0.25, 0.59], *β* = .08. Thus, Hypothesis 2 was not supported.

However, supporting Hypothesis 3 group members in the social identity condition (*M* = 3.04, *SD* = 0.81) interpreted the support they received as significantly more benevolent than participants in the personal identity condition (*M* = 2.83, *SD* = 1.00), *b* = 0.21, *SD* = 0.13, *p* = .048 (one‐tailed), 90% CI [0.002, 0.43], *β* = .23.

## DISCUSSION

The present study examined how social identity shapes social support within and across groups. We tested three hypotheses regarding objective mutual social support across teams (i.e. at the group‐level), as well as perceived social support (provided and received) and perceived benevolence of social support within groups (i.e. at the individual‐level). As predicted, objective mutual social support was greater in groups in the social identity condition than in groups in the personal identity condition. Regarding perceived provided and perceived received support, we did not find any significant differences across experimental conditions. However, in line with our predictions, group members in the social identity condition perceived the support they received as more benevolent than in the personal identity condition.

### Theoretical implications and future research

Our results have several theoretical implications. By examining objective mutual social support at the group‐level—rather than only relying on individual‐level perceptions of support, we take a crucial step towards aligning the operationalization of social support with the theoretical conceptualization of social support within the Social Identity Approach (Tajfel & Turner, 1979; Turner et al., 1987). Thus, our findings extend research by demonstrating that social identity does not merely shape how support is perceived but is observable in patterns of mutual social support within groups. This is in line with the social support hypothesis proposed by Jetten et al. (2017) that a shared social identity should lead to ‘actually giving each other more social support’ (p. 8). Furthermore, the current study supports the notion by Häusser et al. (2020) to better integrate a group‐level approach in social identity research, using the group as a focal entity and as a crucial unit of analysis, thereby demonstrating that mutual support emerges as a group‐level phenomenon when individuals coordinate their actions towards shared goals (Haslam et al., 2005). While previous meta‐analytic evidence has linked aggregated identification on the group‐level to improved health outcomes (Steffens et al., 2017), our finding extends this result by showing that shared social identity also shapes objective mutual social support at the group‐level.

Häusser et al. (2020) further emphasize that social identity processes within groups cannot be fully understood by either focusing on the group‐level or the individual‐level alone. Understanding how social identity shapes mutual social support requires examining group‐ and individual‐level processes in combination. However, we did not find a significant difference in perceived provided and perceived received social support between the experimental conditions. This finding is surprising given the robust association between social identification and perceived social support documented in previous research (e.g. Haslam et al., 2005; Häusser et al., 2023; Junker et al., 2019; Steffens, Cruwys, et al., 2016; Walsh et al., 2015). Yet, considering our finding that groups in the social identity condition engaged in more objective mutual social support, the relationship between social identity and social support appears more nuanced than previously assumed: A shared social identity leads groups to actually give each other more social support during group tasks, but this does not necessarily translate into higher perceived social support of their group members.

One explanation for these diverging results is that perceived social support, as measured in earlier field studies, either reflects appraisals that accumulate over time across multiple events and interactions (Avanzi et al., 2015; Bruner et al., 2021; Drury et al., 2016; Häusser et al., 2023; Procidano & Smith, 1997), or captures support in general terms, without reference to a particular social interaction (Haslam et al., 2005, 2018). Both capture a broader sense of feeling supported rather than distinct supportive behaviour within a specific interaction. Such general assessments leave more room for identity‐based interpretations, as individuals may draw on their identification with the respective group to infer perceived social support. Our assessment of perceived provided and received support focused on specific, concrete supportive behaviours during the task, most of which were informational and practical in nature (e.g. sharing task‐relevant information or ideas; Boyar et al., 2014). These items referenced supportive actions without labelling them as support, rather than whether participants felt supported in the sense of having their needs met. Such behaviour‐specific perceptions may not fully capture the more general or emotional dimensions of social support that are central to broader conceptualizations of perceived support (e.g. ‘Do you get the emotional support you need from other people?’; Haslam et al., 2018; Steffens, Cruwys, et al., 2016).

Moreover, when a shared social identity is salient, supporting one another in a group might be interpreted as normative and thus expected ‘default’ ingroup behaviour rather than as an explicit act of support. The social support hypothesis proposes that shared social identity leads group members ‘to expect to give each other support’ (Jetten et al., 2017, p. 8). This expectation, however, may reflect an anticipation that social support will occur, rather than a heightened perception that more social support has actually been exchanged. Conversely, social support in the absence of a shared social identity might be more noticeable and perceived as deviating from ‘default mode’ precisely because it violates expectations. In this sense, our missing significance for perceived social support might paradoxically reflect the very expectation described by the social support hypothesis: Because group members expected mutual social support, it went unnoticed in their perceptions.

Thus, rather than inflating perceptions of the *extent* of support exchanged, social identity might shape *how* social support is interpreted and evaluated. Haslam et al. (2012) argue that social identity provides a basis for construing support in the spirit in which it is intended, and the social support hypothesis proposes that shared social identity leads individuals to ‘construe received support more positively’ (Jetten et al., 2017, p. 8). Our finding that group members in the social identity condition perceived social support as more benevolent than those in the personal identity condition supports this proposition. More broadly, this aligns with the intergroup attribution literature showing that positive behaviour by ingroup members is more likely to be attributed to internal causes (e.g. genuine concern), whereas the same behaviour by outgroup members is more likely to be attributed to external factors (e.g. social obligation; Hewstone, 1990; Tajfel & Turner, 1979). In this sense, shared social identity might influence the perceived intentionality and sincerity of support rather than merely its frequency. Our results thus highlight the importance of distinguishing between *quantitative* and *qualitative* dimensions of perceived social support: while shared social identity increased mutual social support, it also shaped the relational meaning attached to these behaviours, suggesting that social identity might function as an interpretative lens that structures not only social support within groups but also the evaluative frameworks through which social support is understood and valued. Future research should therefore examine the interpretation of support more broadly, including concrete attribution processes, distinguishing between self‐interested and altruistic motives, and perceptions of the support provider (e.g. perceived warmth or competence).

Lastly, our experimental manipulation of social identity allows for causal inferences. While correlational studies have consistently shown associations between social identification and perceived support (e.g. Haslam et al., 2005; Häusser et al., 2023), experimental evidence for the effect of social identity on social support within groups has been lacking. This is particularly important given evidence that the relationship between identification and support is reciprocal over time (Häusser et al., 2023). The present study extends this work by providing adequately powered causal evidence that making a shared social identity salient increases mutual social support during group interactions.

### Practical implications

Practically, our findings suggest that efforts to enhance team identification and, thereby, social support in groups should address not only the quantity of social support exchanged but also how social support is interpreted or perceived. Even when social support increases, this may not automatically translate into heightened perceptions of support. Attention should thus also be directed to the perceived benevolence of social support. Team‐building activities that foster a shared social identity among group members and emphasize common goals, shared narratives and collective achievements may strengthen the perception that social support stems from genuine concern rather than situational obligation. Additionally, making intentions of social support explicit, for instance, through structured reflection or feedback practices, may help ensure that social support is recognized and attributed to benevolent motives.

Moreover, the present study is the first to successfully adapt established social identity manipulation procedures (Frisch et al., 2015; Häusser et al., 2012) to an online group setting. This is particularly relevant given that virtual teams and remote work environments have become increasingly prevalent, introducing new challenges for effective teamwork as collaboration increasingly depends on digital communication tools (Chinyuku & Qutieshat, 2025). Our findings demonstrate that a shared social identity can be fostered in online settings through relatively simple means, such as visual cues (e.g. a team mascot), assigning a shared team name and emphasizing commonalities among group members. Organizations relying on virtual collaboration would thus benefit from implementing such strategies to create a shared social identity among team members.

### Limitations

The present study is not without limitations. First, participants interacted with previously unknown group members in newly formed groups. In such minimal‐acquaintance settings, shared social identity may function differently than in established groups where members have developed pre‐existing relationships, interaction histories and accumulated knowledge about one another's needs and resources. In established groups, factors such as interpersonal trust, reciprocity norms and role differentiation may interact with shared social identity in shaping mutual social support—processes that cannot emerge in a single initial encounter. Whether shared social identity remains an equally potent driver of mutual social support when these additional relational dynamics are at play, or whether its influence is amplified or attenuated by them, remains an open question.

Second, as stated above, we assessed perceived provided and received support through specific, task‐related supportive actions (Boyar et al., 2014), rather than through more general measures of social support (Haslam et al., 2018; Steffens, Cruwys, et al., 2016). Whether a behaviour, rather than merely noticed, is experienced as support involves a further interpretive judgement—one that a shared social identity may shape even when the behaviour itself is registered similarly across conditions. It is therefore possible that social identity influences perceived support in this more general sense (even though it did not affect reports of the specific behaviours we assessed). Thus, future research should combine behaviour‐specific items with measures of general and more abstract support perceptions to establish which aspects of perceived support are shaped by shared social identity.

Third, we observed social support during a brief group interaction, capturing only a short segment of the social support process. While this approach allowed for controlled observation under standardized conditions, the brief observation window does not allow conclusions about whether the observed effects persist, intensify or diminish as groups develop and members move through different phases of group life. Future research should therefore examine the role of shared social identity in mutual social support within established groups and across extended interaction periods to assess the robustness and generalizability of the present findings.

## CONCLUSION

The present study provides the first experimental evidence that a shared social identity increases objective mutual social support during group interactions and shapes how this support is perceived. By combining behavioural observation with self‐report measures in an online group setting, and using a manipulation of social identity, our findings extend prior research that has relied predominantly on perceived social support and self‐report measures of identification. While a shared social identity increased objective mutual social support within groups and led group members to interpret social support as more benevolent, it did not affect the perceptions of social support. This suggests that the well‐documented association between social identity and perceived social support might at least partly reflect a perceptual identity‐based interpretation. Our findings highlight the importance of distinguishing between quantitative and qualitative dimensions of social support and advance our understanding of group‐level approaches in social identity as a foundation for social support processes.

## AUTHOR CONTRIBUTIONS

**Diana Usmanova:** Conceptualization; methodology; writing – review and editing. **Nina Mareen Junker:** Conceptualization; methodology; formal analysis; writing – review and editing. **Rolf van Dick:** Conceptualization; methodology; writing – review and editing; funding acquisition. **Jan Alexander Häusser:** Conceptualization; methodology; writing – review and editing; supervision; funding acquisition. **Julia Heimrich:** Conceptualization; methodology; formal analysis; visualization; writing – original draft; writing – review and editing.

## FUNDING INFORMATION

This research was supported by a grant by the German Research Foundation (HA 6455/5‐2) awarded to JAH and RvD.

## CONFLICT OF INTEREST STATEMENT

None of the authors has any conflict of interest.

## ETHICS STATEMENT

At the institutions where this research was conducted ethical clearance is not mandatory for all studies. This research uses a non‐invasive survey design with established questionnaires only. It was therefore exempt from ethical clearance. The research was conducted in line with the ethical guidelines of the German Psychological Society and the Declaration of Helsinki.

## Supporting information


Data S1.

**Appendix A.** Full workplace simulation.

## Data Availability

Pre‐registrations for our Pilot study (https://aspredicted.org/g6p7‐2pmj.pdf) as well as our Main study (https://aspredicted.org/fsp6‐xphx.pdf) are available online. Data, codebook and study materials are available on the OSF platform (https://osf.io/y6utk/overview?view_only=c658b464beaa4144b9e3843c1fc96dfb).

## Footnotes

^1^
Because objective mutual social support within groups constitutes a reciprocal exchange in which providing and receiving support are inherently linked, we examine both facets within a single hypothesis. Further, during the revision process we slightly changed the formulation of the hypotheses from what was pre‐registered to improve clarity. The content of the hypotheses remains the same.

^2^
Zoom is a widely used video conferencing platform that enables real‐time audio and video communication between participants in separate locations, allowing for face‐to‐face‐like interaction in a fully remote setting.

^3^
As the study was part of a larger research project, additional measures of stress, team satisfaction, personality, collective efficacy and trust were also collected. We pre‐registered an additional hypothesis regarding collective self‐efficacy (H4) that is beyond the scope of the current paper. We report those results in the online supplement material.

^4^
Due to a survey programming error, perceived provided social support was measured on a 6‐point scale instead of the intended 7‐point scale. Scores were linearly rescaled to a 1–7 metric to ensure comparability.
