# Supplementary material for: The effect of social identity on objective and perceived social support in groups
Source: Br J Soc Psychol. 2026 Sep 8;65(4):e70130. doi: 10.1111/bjso.70130 (PMC13554828; doi:10.1111/bjso.70130)
Supplement: Supplementary file 1 [file BJSO-65-0-s001.docx]

**Online Supplement for the Manuscript “The Effect of Social Identity on Objective and Perceived Social Support in Groups”**

## Results of additional Hypothesis regarding Collective Self-Efficacy

## We pre-registered an additional hypothesis (H4) regarding collective self-efficacy, stating that in the social identity condition, collective self-efficacy is higher than in the personal identity condition. Collective self-efficacy was measured using two items developed by van Dick et al. (1999). Participants rated their agreement with the items (sample item: “We can achieve difficult goals in this group”) on a scale from 1 = *Do not agree at all* to 7 = *Fully agree*. The correlation of the two items was .87. Results show that group members in the social identity condition (M = 5.40, SD = 1.35) perceived collective self-efficacy is not higher than participants in the personal identity condition (*M* = 5.10, *SD* = 1.51), *b* = .31, *SD* = 0.22, *p* = .091 (one-tailed), 90% CI [-0.64,0.66], *β* = 0.17. Thus, this additional hypothesis is not supported.
