# Supplementary material for: The effect of social identity on objective and perceived social support in groups
Source: Br J Soc Psychol. 2026 Sep 8;65(4):e70130. doi: 10.1111/bjso.70130 (PMC13554828; doi:10.1111/bjso.70130)
Supplement: Supplementary file 2 [file BJSO-65-0-s002.docx]

# Appendix A

**Employee 1:**

1. Your supervisor requires a table of the business performance of a competing airline to find out how it has developed over the past year. Fill in the table below using the attached financial report (APPENDIX pages 3-5). Additionally should the total weekly costs of the new flight destinations also be included in the table so that your supervisor can incorporate them into the next meeting to discuss future costs.
2. Your task is to conduct a brief customer satisfaction analysis for a new flight route from Frankfurt (FRA) to Barcelona (BCN). You have received a total of 15 customer feedback entries that you need to summarize and evaluate.

**Employee 2:**

3. Your supervisor has tasked you with developing a new in-flight menu for the flight route from Frankfurt (FRA) to Beijing (PEK). The menu should accommodate various culinary preferences and be attractively designed. Remember that one starter, main course, and dessert each should be suitable for vegetarians.
4. You are responsible for the shift schedule for next week. You need to assign the employees listed below. Since employee satisfaction is important to you, you want to design the schedule so that all individual employee preferences are accommodated. The schedule is divided into three shifts. The morning shift runs from 6:00 AM to 12:00 PM, the afternoon shift from 12:00 PM to 6:00 PM, and the evening shift from 6:00 PM to 12:00 AM. Each of your employees normally has two days off per week and all shifts must be covered.

**Employee 3:**

5. Your task is to conduct a cost analysis for new flight routes from Frankfurt (FRA) to 7 international destinations. You need to consider various cost factors such as fuel costs, personnel, airport fees, and maintenance costs. Each route is to be operated 5 times per week. Enter the individual costs for each cost factor in the table and calculate the total costs for each destination per week.
6. Your task is to create the flight schedule for the upcoming week (Monday to Sunday). You need to consider various factors such as outbound and return flights, the number of flights per day, the flight destinations, the availability of aircraft, and the appropriate employee to oversee each flight.

**Employee 1:**

1. **Your supervisor requires a table of the business performance of a competing airline to find out how it has developed over the past year. Fill in the table below using the attached financial report (LINK). Additionally, the total weekly costs of the new flight destinations should be included in the table so that your supervisor can incorporate them into the next meeting to discuss future costs.**

| **Key Figures** |  |  |  |
| --- | --- | --- | --- |
|  |  | **Jan-Mar 2024** | **Jan-Mar 2023** |
| **Revenue and Results** |  |  |  |
| Revenue | M € |  |  |
| Adjusted free Cash Flow | M € |  |  |
| Adjusted EBIT | M € |  |  |
| **Balance Sheet and Cash Flow Key Figures** | |  |  |
| Operating Expenses | M € |  |  |
| Net Credit Debt | M € |  |  |
| Operating Material Costs | M € |  |  |
| **Services and Fees** |  |  |  |
| Fees | M € |  |  |
| Raw, Auxiliary, and Operating Materials | M € |  |  |
| Passenger Services | M € |  |  |
| **Costs New Flight Destinations** |  | **Per Week** |  |
| Tokyo | € |  |  |
| New York | € |  |  |
| Dubai | € |  |  |
| Rio de Janeiro | € |  |  |
| Sydney | € |  |  |
| London | € |  |  |

2. **Your task is to conduct a brief customer satisfaction analysis for a new flight route from Frankfurt (FRA) to Barcelona (BCN). You have received a total of 15 customer feedback entries that you need to summarize and evaluate.**

Collection and Evaluation of Customer Feedback:

- Read the following customer feedback for the new flight route:
  - "The flight was on time and the cabin crew was very friendly. However, the seats could be more comfortable. I had back pain after the flight."
  - „On my flight to Shanghai (China), I had a great selection of movies. It would have been nice if the food service had offered a fruity dessert option, as the menu was very heavy“.
  - "I was impressed by the cleanliness and service on board. However, the food was really not good and I barely touched it. It would be nice if there were more options."
  - "The check-in process was quick and efficient, but there was no entertainment during the flight. I had expected to have at least a few movies to choose from. It was a bit boring."
  - "Good value for money, but the waiting times during boarding were too long. It would be helpful if boarding were better organized. Nevertheless, the staff was very friendly."
  - "Very pleasant flight, but the Wi-Fi on board did not work well. I could barely establish a connection. This was particularly annoying because I wanted to work during the flight."
  - "The flight was pleasant, but the food selection was limited. There was no vegetarian option on a flight to China in business class, so I would like more options for vegans and vegetarians.
  - "Friendly and helpful staff, but the air conditioning was set too cold. I was freezing during the flight. Perhaps blankets could be provided."
  - "Punctual departure and arrival, but the meals on board were disappointing. The food was cold and tasteless. I hope this will be improved in the future."
  - "Easy check-in and clean aircraft, but the Wi-Fi was slow. I could barely do anything online. Faster internet would be great."
  - "Good in-flight entertainment, but the seat comfort was unsatisfactory. The seats were hard and uncomfortable. It was difficult to relax."
  - "Excellent customer service, but the waiting time during boarding was too long. It took far too long for all passengers to board. A more efficient method would be helpful."
  - "Very good flight, but the drinks were too expensive. I had to pay a high price for a simple juice. Slightly cheaper options would be better."
  - "Efficient boarding, but the selection of in-flight meals was limited. There were only a few options and no vegetarian alternatives. A wider selection would be desirable."
  - "Friendly staff, but the seats were too cramped. There was hardly any legroom and it was uncomfortable. More space would be desirable."
  - „On my flight to Beijing, I would have wished for the menu to offer a greater variety of traditional Chinese dishes to better reflect cultural authenticity and the travel experience. The flight attendant was nice but could not offer me any other options.“
  - "Good value for money, but the Wi-Fi did not work properly. I could barely establish a connection. This was frustrating because I wanted to work."

*Instructions:*

Summarize the most common positive and negative aspects of the customer feedback in bullet points and make suggestions for improvement based on them.

POSITIVE:

NEGATIVE:

SUGGESTIONS FOR IMPROVEMENT:

**Employee 2:**

3. **Your supervisor has tasked you with developing a new in-flight menu for the flight route from Frankfurt (FRA) to Beijing (PEK). The menu should accommodate various culinary preferences and be attractively designed. Also take customer feedback into account. There have been several requests and suggestions for improvement in recent weeks.**

**Menu Theme:** Come up with a theme for the in-flight menu that suits the route (e.g., 'Mediterranean Delicacies')

**Food and Beverages:** Select three starters, three main courses, and three desserts to be offered on the flight.

**Menu Description:** Write a short, appetizing description for each dish

.

Theme:

Starter 1:

Starter 2:

Starter 3:

Main Course 1:

Main Course 2:

Main Course 3:

Dessert 1:

Dessert 2:

Dessert 3:

4. **You are responsible for the shift schedule for next week. You need to assign the employees listed below. Since employee satisfaction is important to you, you want to design the schedule so that all individual employee preferences are accommodated. The schedule is divided into three shifts. The morning shift runs from 6:00 AM to 12:00 PM, the Mittags-Schicht from 12:00 PM to 6:00 PM, and the Abend-Schicht from 6:00 PM to 12:00 AM. Each of your employees normally has two days off per week and all shifts must be covered.**

**Thomas**, an experienced employee who is always punctual and reliable, loves the morning hours. As a passionate jogger, he enjoys running a lap through the park before work and going straight to the office. Thomas would like to have Tuesdays and Fridays off to care for his sick mother, who has her therapy sessions on those days. Additionally, he would like to work early on weekends to have the afternoon free. He also considers it important to have Sundays off to recover from the demanding week and spend time with his girlfriend..

**Anna**, a dedicated young woman who is studying business administration on the side, appreciates the late shifts since she attends lectures and seminars during the day. Anna would like to have Mondays and Fridays off to complete her coursework and prepare for exams. On Sundays, she volunteers at a hospital.

**Lucas**, an enthusiastic athlete and member of a local basketball team, prefers the midday shift, which allows him to train in the morning and participate in games and training sessions in the evening. Lukas would like Wednesdays and Saturdays off to attend regular team meetings and tournaments. On Tuesdays, he is always on field duty and therefore cannot come to the office. To support his colleagues, Lukas has agreed to take the early shift once per week.

**Marie**, a creative soul with a passion for painting, is flexible regarding her shifts but has a strong preference for working on Sundays. She likes to use Sundays to complete her tasks in the quiet atmosphere of the office and dedicate the rest of the week to her artistic projects and workshops. Marie would like Wednesdays, Fridays, and Saturdays off to devote more time to her art and participate in various exhibitions.

**Peter**, an early riser and family man, prefers the early shift since he can then spend the afternoon and evening with his children. Peter would like Mondays and Thursdays off to support his wife, who is very busy with work on those days. He also does not want to work the same shift on two consecutive days, as he values the variety and the opportunity to get to know different tasks and colleagues. Peter takes the evening shift once a week to relieve Anna and Marie and to ensure that all shifts are evenly staffed.

|  | **Monday** | **Tuesday** | **Wednesday** | **Thursday** | **Friday** | **Saturday** | **Sunday** |
| --- | --- | --- | --- | --- | --- | --- | --- |
| **6:00-13:00** |  |  |  |  |  |  |  |
| **12:00-19:00** |  |  |  |  |  |  |  |
| **18:00-00:00** |  |  |  |  |  |  |  |

**Employee 3:**

5. **Your task is to conduct a cost analysis for new flight routes from Frankfurt (FRA) to 7 international destinations. You need to consider various cost factors such as fuel costs, personnel, airport fees, and maintenance costs. Each route is to be operated 5 times per week. Enter the individual costs for each cost factor in the table and calculate the total costs for each destination per week.**

Destinations: New York (JFK), Tokyo (HND), Dubai (DXB), Sydney (SYD), Rio de Janeiro (GIG), Johannesburg (JNB), and London (LHR)

Costs vary depending on the flight destination due to different distances and regional fees:

- **New York (JFK):**
  - Fuel costs: 10.000 EUR per flight
  - Personnel costs: 4.000 EUR per flight
  - Airport fees: 3.000 EUR per flight
  - Maintenance costs: 2.000 EUR per flight
- **Tokyo (HND):**
  - Fuel costs: 15.000 EUR per flight
  - Personnel costs: 5.000 EUR per flight
  - Airport fees: 4.000 EUR per flight
  - Maintenance costs: 3.000 EUR per flight
- **Dubai (DXB):**
  - Fuel costs: 8.000 EUR per flight
  - Personnel costs: 3.500 EUR per flight
  - Airport fees: 2.500 EUR per flight
  - Maintenance costs: 2.000 EUR per flight
- **Sydney (SYD):**
  - Fuel costs: 18.000 EUR per flight
  - Personnel costs: 6.000 EUR per flight
  - Airport fees: 5.000 EUR per flight
  - Maintenance costs: 4.000 EUR per flight
- **Rio de Janeiro (GIG):**
  - Fuel costs: 12.000 EUR per flight
  - Personnel costs: 4.500 EUR per flight
  - Airport fees: 3.500 EUR per flight
  - Maintenance costs: 2.500 EUR per flight
- **Johannesburg (JNB):**
  - Fuel costs: 14.000 EUR per flight
  - Personnel costs: 5.000 EUR per flight
  - Airport fees: 4.000 EUR per flight
  - Maintenance costs: 3.000 EUR per flight
- **London (LHR):**
  - Fuel costs: 6.000 EUR per flight
  - Personnel costs: 3.000 EUR per flight
  - Airport fees: 2.000 EUR per flight
  - Maintenance costs: 1.500 EUR per flight

| Destination | Fuel Costs (per flight) | Personnel Costs (per flight) | Airport Fees (per flight) | Maintenance Costs (per flight) | Flights per Week | Total Costs |
| --- | --- | --- | --- | --- | --- | --- |
| JFK |  |  |  |  | 5 |  |
| HND |  |  |  |  | 5 |  |
| DXB |  |  |  |  | 5 |  |
| SYD |  |  |  |  | 5 |  |
| GIG |  |  |  |  | 5 |  |
| JNB |  |  |  |  | 5 |  |
| LHR |  |  |  |  | 5 |  |

6. **Your second task is to create the flight schedule for the upcoming week (Monday to Sunday). You need to consider various factors such as outbound and return flights, the number of flights per day, the flight destinations, the availability of aircraft, and the appropriate employee to oversee each flight.**

**Flight Destinations and Number of Flights:**

- Our airline operates daily flights to the following destinations:
  - Frankfurt (FRA) nach Berlin (BER): 1h
  - Frankfurt (FRA) nach Porto (OPO): 3h
  - Frankfurt (FRA) nach OSLO (OSL): 2h
  - Frankfurt (FRA) nach München (MUC): 30min
- At least two flights per day must be scheduled for each destination, and the times for the different destinations must vary each day.

**Aircraft Availability:**

- We have a total of 4 aircraft available:
  - Aircraft A: Airbus A320 (200 seats)
  - Aircraft B: Boeing 737 (180 seats)
  - Aircraft C: Airbus A319 (150 seats)
  - Aircraft D: Embraer E190 (100 seats)
- Each aircraft may operate a maximum of two flights per day.

**Compliance with Departure and Arrival Times:**

- Flights may take place between 6:00 AM and 12:00 AM.
- At least one hour must be scheduled between each flight for preparation and refueling (This does not apply to the first and last flight of the day)

**Employees:**

- Thomas, Anna, Lucas, Marie, Peter
- All employees have different working hours (see Task 4)

*Instructions:*

- Enter the planned flights for each day of the week in the table. Add rows if needed.
- Make sure that all conditions are met (number of flights per destination, aircraft availability, compliance with times, appropriate employee).Check your planning for plausibility and make sure that no overlaps or errors occur.

| Day | Departure Time | Arrival Time | Departure Airport | Arrival Airport | Aircraft Type | Employee |
| --- | --- | --- | --- | --- | --- | --- |
| Monday |  |  |  |  |  |  |
| Monday |  |  |  |  |  |  |
| … |  |  |  |  |  |  |
| Tuesday |  |  |  |  |  |  |
| Tuesday |  |  |  |  |  |  |
| … |  |  |  |  |  |  |
| Wednesday |  |  |  |  |  |  |
| Wednesday |  |  |  |  |  |  |
| … |  |  |  |  |  |  |
| Thursday |  |  |  |  |  |  |
| Thursday |  |  |  |  |  |  |
| … |  |  |  |  |  |  |
| Friday |  |  |  |  |  |  |
| Friday |  |  |  |  |  |  |
| … |  |  |  |  |  |  |
| Saturday |  |  |  |  |  |  |
| Saturday |  |  |  |  |  |  |
| … |  |  |  |  |  |  |
| Sunday |  |  |  |  |  |  |
| Sunday |  |  |  |  |  |  |
| …. |  |  |  |  |  |  |
